# Supplementary material for: Spatially informed clustering, integration, and deconvolution of spatial transcriptomics with GraphST
Source: Nat Commun. 2023 Mar 1;14:1155. doi: 10.1038/s41467-023-36796-3 (PMC9977836; doi:10.1038/s41467-023-36796-3)
Supplement: Supplementary file 2 — Reporting Summary [file 41467_2023_36796_MOESM2_ESM.pdf]

nature portfolio

Corresponding author(s): Jinmiao Chen

Last updated by author(s): Feb 7, 2023

# Reporting Summary

Nature Portfolio wishes to improve the reproducibility of the work that we publish. This form provides structure for consistency and transparency in reporting. For further information on Nature Portfolio policies, see our Editorial Policies and the Editorial Policy Checklist.

## Statistics

For all statistical analyses, confirm that the following items are present in the figure legend, table legend, main text, or Methods section.

| n/a | Confirmed | |
|---|---|---|
| ☐ | ☒ | The exact sample size (*n*) for each experimental group/condition, given as a discrete number and unit of measurement |
| ☐ | ☒ | A statement on whether measurements were taken from distinct samples or whether the same sample was measured repeatedly |
| ☐ | ☒ | The statistical test(s) used AND whether they are one- or two-sided<br>*Only common tests should be described solely by name; describe more complex techniques in the Methods section.* |
| ☐ | ☒ | A description of all covariates tested |
| ☐ | ☒ | A description of any assumptions or corrections, such as tests of normality and adjustment for multiple comparisons |
| ☐ | ☒ | A full description of the statistical parameters including central tendency (e.g. means) or other basic estimates (e.g. regression coefficient) AND variation (e.g. standard deviation) or associated estimates of uncertainty (e.g. confidence intervals) |
| ☐ | ☒ | For null hypothesis testing, the test statistic (e.g. *F*, *t*, *r*) with confidence intervals, effect sizes, degrees of freedom and *P* value noted<br>*Give P values as exact values whenever suitable.* |
| ☒ | ☐ | For Bayesian analysis, information on the choice of priors and Markov chain Monte Carlo settings |
| ☐ | ☒ | For hierarchical and complex designs, identification of the appropriate level for tests and full reporting of outcomes |
| ☒ | ☐ | Estimates of effect sizes (e.g. Cohen's *d*, Pearson's *r*), indicating how they were calculated |

*Our web collection on statistics for biologists contains articles on many of the points above.*

## Software and code

Policy information about availability of computer code

| | |
|---|---|
| Data collection | No software was used for data collection. |
| Data analysis | GraphST v1.1.1 (https://github.com/JinmiaoChenLab/GraphST), Seurat v4.1.1 (https://github.com/satijalab/seurat), Giotto v1.1.0 (https://github.com/RubD/Giotto), SpaGCN v1.2.0 (https://github.com/jianhuupenn/SpaGCN), BayesSpace v1.5.1 (https://github.com/edward130603/BayesSpace), SpaceFlow v1.0.0 (https://github.com/hongleir/SpaceFlow), conST v1.0.0 (https://github.com/ys-zong/conST) and STAGATE v1.0.1 (https://github.com/QIFEIDKN/STAGATE) were used for spatial domain identification. GraphST v1.1.1 (https://github.com/JinmiaoChenLab/GraphST), SpaGCN v1.2.0 (https://github.com/jianhuupenn/SpaGCN), Harmony v1.0.0 (https://github.com/immunogenomics/harmony), scVI v0.19.0 (https://github.com/scverse/scvi-tools), and STAGATE v1.0.1 (https://github.com/QIFEIDKN/STAGATE) were used for multiple tissue analysis. GraphST v1.1.1 (https://github.com/JinmiaoChenLab/GraphST), cell2location v1.0.1 (https://github.com/BayraktarLab/cell2location), RCTD v1.2.0 (https://github.com/dmcable/spacexr), Seurat v4.0.5 (https://github.com/satijalab/seurat), SPOTlight v0.1.7 (https://github.com/maciejkula/spotlight), and NNSL v1.0.4 (https://github.com/theislab/AutoGeneS) were used for ST and scRNA data integration, i.e., deconvolution. Scanpy v1.9.1 was used for data pre-processing and result visualization. mclust v6.0.0 was used for result visualization. |

For manuscripts utilizing custom algorithms or software that are central to the research but not yet described in published literature, software must be made available to editors and reviewers. We strongly encourage code deposition in a community repository (e.g. GitHub). See the Nature Portfolio guidelines for submitting code & software for further information.

## Data

Policy information about availability of data

All manuscripts must include a data availability statement. This statement should provide the following information, where applicable:

- Accession codes, unique identifiers, or web links for publicly available datasets
- A description of any restrictions on data availability
- For clinical datasets or third party data, please ensure that the statement adheres to our policy

We analyzed multiple spatial transcriptomics datasets for spatial clustering, spatial transcriptomics data integration, and scRNA and spatial transcriptomics data integration. Publicly available data were downloaded from the following websites or accession numbers:
1. LIBD human dorsolateral prefrontal cortex (Maynard et al. 2021)
Figures 2A, C, Figure 5F, Supplementary Figures S1, S7, S8, S14, and S15. The count matrix and spatial data can be downloaded from http://research.libd.org/spatialLIBD/.
2. Mouse brain 10X data
Figure 4G, Supplementary Figures S2, S9, S10, and S11. The count matrix and spatial data can be downloaded from https://www.10xgenomics.com/resources/datasets.
3. Mouse olfactory bulk Stereo-seq data (Chen et al. 2022)
Figures 2D-F. The count matrix and spatial data can be downloaded from https://drive.google.com/drive/folders/1RixFo9MdX3fpj_cmrZiRxlBdoD2DTep4.
4. Mouse hippocampus Slide-seqV2 data
Figures 2G-I. The count matrix and spatial data can be downloaded from https://portals.broadinstitute.org/single_cell/study/slide-seq-study.
5. Mouse embryo Stereo-seq data (Chen et al. 2022)
Figures 3A-G, Supplementary Figures S3 and S4. The count matrix and spatial data can be downloaded from https://db.cngb.org/stomics/mosta/.
6. Simulated data for predicting spatial distribution of scRNA-seq data
Figure 5A. The simulated data can be downloaded from https://github.com/QuKunLab/SpatialBenchmarking/tree/main/FigureData/Figure4.
7. Human lymph node data for deconvolution (Kleshchevnikov et al. 2022)
Figures 5B-E, Supplementary Figures S5 and S6. Both spatial transcriptomics and scRNA-seq data were obtained from Kleshchevnikov et al. 2022 and can be downloaded from https://drive.google.com/drive/folders/1ns-EsWBu-SNrJ39j-q-AFIV5U-aXFwXf.
8. scRNA-seq reference data for mouse anterior brain deconvolution
Supplementary Figures S9-11. scRNA-seq data can be downloaded from https://portal.brain-map.org/atlases-and-data/rnaseq/mouse-whole-cortex-and-hippocampus-10x.
9. scRNA-seq reference data for DLPFC 151673 slice deconvolution
Figure 5F, Supplementary Figures S7 and S8. scRNA-seq data can be downloaded from https://www.ncbi.nlm.nih.gov/geo/query/acc.cgi?acc=GSE144136.
10. Human breast cancer 10X data
Figure 6, Supplementary Figure S12. The count matrix and spatial data can be downloaded from https://www.10xgenomics.com/resources/datasets/human-breast-cancer-block-a-section-1-1-standard-1-1-0. scRNA-seq data for deconvolution can be downloaded from database DISCO (https://www.immunesinglecell.org/).

The data used in this study has been uploaded to Zenodo and is freely available at: https://zenodo.org/record/6925603#.YuM5WXZBwuU. A summary of the datasets is available in the Tables S1 and S2.

An open-source Python implementation of the GraphST toolkit is accessible at https://github.com/JinmiaoChenLab/GraphST.

## Human research participants

Policy information about studies involving human research participants and Sex and Gender in Research.

| | |
|---|---|
| Reporting on sex and gender | N.A. |
| Population characteristics | N.A. |
| Recruitment | N.A. |
| Ethics oversight | N.A. |

Note that full information on the approval of the study protocol must also be provided in the manuscript.

# Field-specific reporting

Please select the one below that is the best fit for your research. If you are not sure, read the appropriate sections before making your selection.

☒ Life sciences ☐ Behavioural & social sciences ☐ Ecological, evolutionary & environmental sciences

For a reference copy of the document with all sections, see nature.com/documents/nr-reporting-summary-flat.pdf

# Life sciences study design

All studies must disclose on these points even when the disclosure is negative.

| | |
|---|---|
| Sample size | We used publicly available data in all figures except Figure 4A-E. To generate data presented in Figure 4A-E, we harvested breast tumor samples from two untreated mice NC3 and NC4. From each tumor, we acquired two serial sections that gave us a total sample size of 4. |
| Data exclusions | All spots and genes were used, no exclusion was done prior to analysis. |
| Replication | We have 4 replicates for untreated breast tumors. |
| Randomization | This is not relevant as we have only one group, i.e., untreated breast tumor. |
| Blinding | This is not relevant as we have only one group, i.e., untreated breast tumor. |

# Reporting for specific materials, systems and methods

We require information from authors about some types of materials, experimental systems and methods used in many studies. Here, indicate whether each material, system or method listed is relevant to your study. If you are not sure if a list item applies to your research, read the appropriate section before selecting a response.

## Materials & experimental systems

| n/a | Involved in the study |
|---|---|
| ☒ | ☐ Antibodies |
| ☐ | ☒ Eukaryotic cell lines |
| ☒ | ☐ Palaeontology and archaeology |
| ☐ | ☒ Animals and other organisms |
| ☒ | ☐ Clinical data |
| ☒ | ☐ Dual use research of concern |

## Methods

| n/a | Involved in the study |
|---|---|
| ☒ | ☐ ChIP-seq |
| ☒ | ☐ Flow cytometry |
| ☒ | ☐ MRI-based neuroimaging |

## Eukaryotic cell lines

Policy information about cell lines and Sex and Gender in Research

| | |
|---|---|
| Cell line source(s) | 4T1 cells from ATCC (mouse mammary cancer). |
| Authentication | No authentication. |
| Mycoplasma contamination | No mycoplasma contamination. |
| Commonly misidentified lines (See ICLAC register) | N.A. |

## Animals and other research organisms

Policy information about studies involving animals; ARRIVE guidelines recommended for reporting animal research, and Sex and Gender in Research

| | |
|---|---|
| Laboratory animals | Mice BALB/c NTAC 20 weeks. |
| Wild animals | N.A. |
| Reporting on sex | Female mice were used as the cells injected were mammary cancer cells. |
| Field-collected samples | N.A. |
| Ethics oversight | All animal work was approved by the NUS Institutional Animal Care and Use Committee (IACUC) and was in accordance with the National Advisory Committee for laboratory Animal Research (NACLAR) Guidelines (Guidelines on the Care and Use of Animals for Scientific Purposes). Protocol approval R18-0635. |

Note that full information on the approval of the study protocol must also be provided in the manuscript.
